# Memory Systems and the Addicted Brain

**DOI:** 10.3389/fpsyt.2016.00024

**Published:** 2016-02-25

**Authors:** Jarid Goodman, Mark G. Packard

**Affiliations:** ^1^Department of Psychology, Texas A&M Institute for Neuroscience, Texas A&M University, College Station, TX, USA

**Keywords:** memory, drug addiction, hippocampus, striatum, amygdala, stress, anxiety

## Abstract

The view that anatomically distinct memory systems differentially contribute to the development of drug addiction and relapse has received extensive support. The present brief review revisits this hypothesis as it was originally proposed 20 years ago (1) and highlights several recent developments. Extensive research employing a variety of animal learning paradigms indicates that dissociable neural systems mediate distinct types of learning and memory. Each memory system potentially contributes unique components to the learned behavior supporting drug addiction and relapse. In particular, the shift from recreational drug use to compulsive drug abuse may reflect a neuroanatomical shift from cognitive control of behavior mediated by the hippocampus/dorsomedial striatum toward habitual control of behavior mediated by the dorsolateral striatum (DLS). In addition, stress/anxiety may constitute a cofactor that facilitates DLS-dependent memory, and this may serve as a neurobehavioral mechanism underlying the increased drug use and relapse in humans following stressful life events. Evidence supporting the multiple systems view of drug addiction comes predominantly from studies of learning and memory that have employed as reinforcers addictive substances often considered within the context of drug addiction research, including cocaine, alcohol, and amphetamines. In addition, recent evidence suggests that the memory systems approach may also be helpful for understanding topical sources of addiction that reflect emerging health concerns, including marijuana use, high-fat diet, and video game playing.

## Introduction

Investigators often look to mechanisms of learning and behavior to explain how human psychopathology is acquired and expressed. An example of such an application was provided by Norman M. White who employed tenets of classical learning theory and experimental evidence supporting the existence of multiple memory systems in the brain to provide a novel, influential approach to drug addiction (1). Specifically, White indicated that drugs can play the part of “reinforcers” that, like food or water in a learning task, strengthen associations among drug-related stimuli, context, and behavior to promote drug taking and, over time, addiction. White also incorporated the emerging hypothesis that there are different types of memory that are mediated by dissociable neural systems. According to this novel view, drugs can directly modulate multiple neural systems, and these neural systems go onto encode distinct components of the drug-related memory that, when expressed, promote further drug taking.

The year 2016 marks the 20th anniversary of the multiple memory systems view of drug addiction as described by White. The present review revisits this influential hypothesis, while highlighting some important recent developments that have not only substantiated the original hypothesis but have also produced additional insights into how multiple memory systems potentially support drug addiction.

## The Multiple Memory Systems View of Addiction

Converging evidence from studies employing humans and lower animals indicates that mammalian memory is mediated by relatively independent neural systems [for reviews, see Ref. (2–4)]. The early experiments dissociating multiple memory systems were primarily conducted in the radial maze and indicated unique mnemonic functions for the hippocampus, dorsal striatum, and amygdala (5, 6). The hippocampus mediates a cognitive/spatial form of memory, whereas the dorsal striatum mediates stimulus–response (S–R) habit memory. The amygdala mediates Pavlovian and stimulus-affect-associative relationships (6, 7), while also subserving the modulatory role of emotional arousal on other types of memory (8–12).

**Figure 1:**
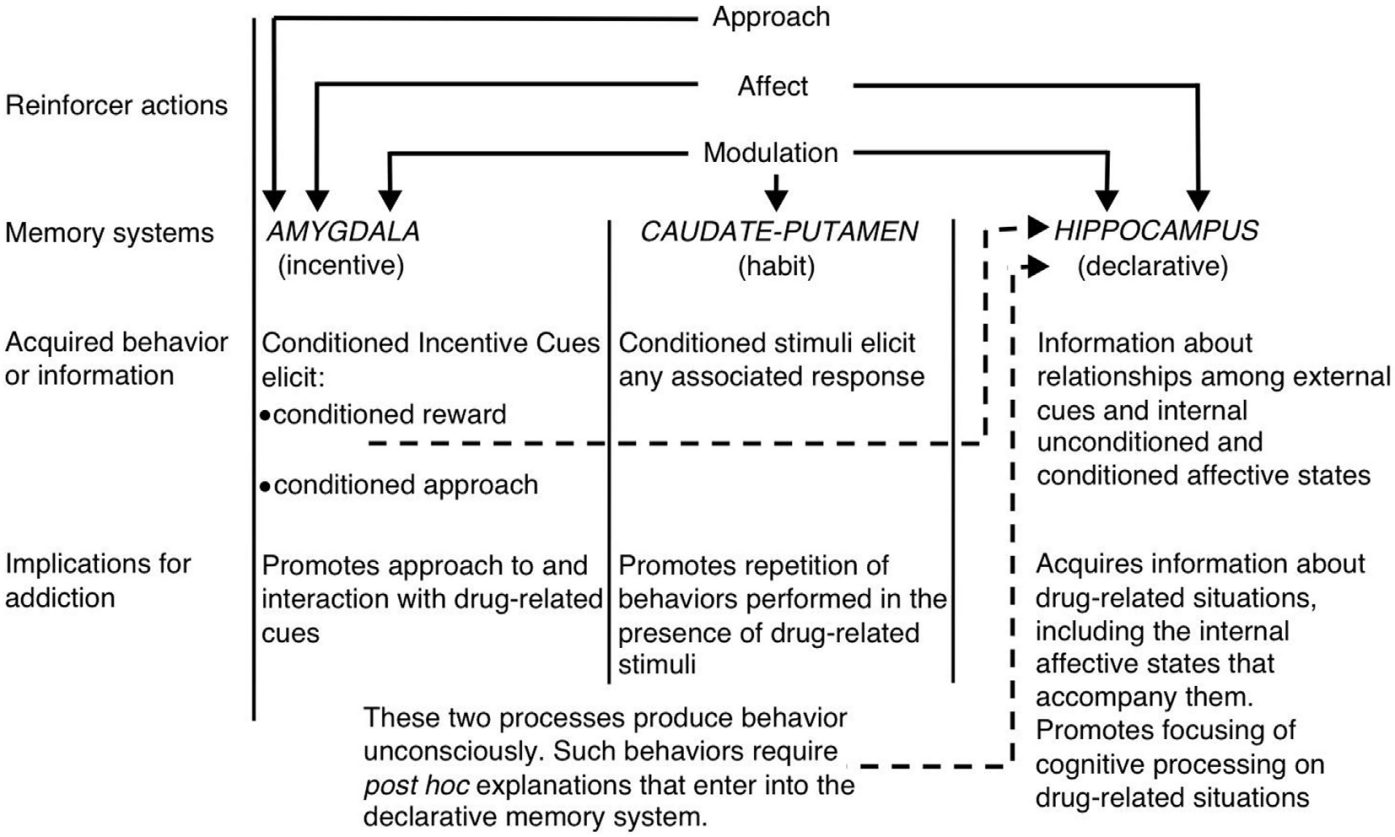
**White’s (1) multiple memory systems view of drug addiction**. Like natural reinforcers, addictive drugs possess several “reinforcer actions,” including the ability to invoke positive/negative affect, approach, and modulation of memory systems. The amygdala, caudate–putamen (i.e., dorsal striatum), and hippocampus mediate dissociable memory systems, and each memory system presumably encodes unique components of drug-related memories. Given their memory modulatory properties, addictive drugs can potentially enhance their own self-administration by enhancing the function of these systems. (Reprinted from White with permission from John Wiley & Sons.)

Within the context of the multiple systems view of memory, White (1) suggested that the hippocampus, dorsal striatum, and amygdala encode unique components of drug-related memories (see Figure 1). The hippocampus encodes explicit knowledge pertaining to the relationship between cues and events (i.e., stimulus–stimulus associations) in the drug context. Importantly, the hippocampus does not encode behavioral responses, but rather the information acquired by the hippocampus can be used to generate the appropriate behavioral responses to receive drug reinforcement. On the other hand, the dorsal striatum encodes associations between drug-related stimuli and behavioral responses. This may allow the presentation of a drug-related cue to activate an automatic behavioral response that results in drug taking (e.g., running approach or instrumental lever press). The amygdala encodes Pavlovian-associative relationships, thus allowing neutral cues in the drug context to become associated with the drug reward. Animals later react to these conditioned cues similarly to how they originally reacted to the drug. Specifically, the conditioned cues activate conditioned emotional responses, including internal affective states and conditioned approach toward (or in some cases avoidance from) the conditioned cue. Another critical component of White’s hypothesis is that drugs can modulate memory function of each of these brain regions. Thus, drugs can potentially enhance their own self-administration via augmenting consolidation of the drug-related memories encoded by the hippocampus, amygdala, and dorsal striatum (see Figure 1).

Consistent with the multiple memory systems view of drug addiction, extensive evidence indicates critical roles for the hippocampus, dorsal striatum, and amygdala in drug addiction and relapse for a variety of abused substances [for review, see Ref. (13)]. The dorsal hippocampus appears to have a role in the contextual control of drug seeking for cocaine (14–16). The lateral region of the dorsal striatum (DLS) mediates S–R habitual lever pressing for cocaine and alcohol (17, 18), and the basolateral amygdala (BLA) mediates conditioned drug seeking for cocaine, alcohol, and heroin (19–22). Also consistent with White’s hypothesis, substances of abuse can modulate the mnemonic functions of the hippocampus, dorsal striatum, and amygdala (23–31).

Recent studies have contributed novel amendments to the multiple memory systems approach to drug addiction. Key features of this contemporary view include (1) a neuroanatomical shift over time to DLS-dependent habit memory, (2) competitive interactions between memory systems, (3) the role of stress and anxiety in enhancing habitual drug seeking, and (4) the application of this hypothesis to new emerging sources of addiction.

## The Neuroanatomical Shift from Cognition to Habit

In experimental learning situations, subjects typically employ purposeful behavior when initially solving a task. However, following extensive training, behavior becomes autonomous and can be performed with little attention, intention, or cognitive effort, constituting a “habit” [for review, see Ref. (32)]. In early demonstrations of this shift from cognitive control of behavior to habit, rodents were trained using food reward in a dual-solution plus-maze task (33–35). In this task, rats were released from the same starting position (e.g., the south arm) and had to make a consistent body-turn at the maze intersection to receive food reward always located in the same goal arm (e.g., always make a left turn to find food in the west arm). Rats could solve this task by either learning a consistent body-turn response or by making whatever response necessary to go the same spatial location. To determine which strategy the rats employed, investigators implemented a probe test in which animals were released from the opposite start arm (e.g., the north arm). If animals made the opposite body-turn to go the original goal location, they were identified as place learners. If animals made the same body-turn as during training (i.e., going to the arm opposite to the original goal location), animals were identified as response learners. Evidence indicates that after some training, most animals display place learning, whereas after extensive training, animals shift to habitual response learning (34–36). Interestingly, this shift from place learning to response learning may reflect a neuroanatomical shift. The initial use of place learning in this task is mediated by the hippocampus and dorsomedial striatum [DMS (36, 37)], whereas the use of response learning after extended training is mediated by the DLS (36).

In addition to early demonstrations using the plus-maze (34, 35), the behavioral shift to habit memory was later demonstrated using operant lever pressing paradigms (38–42). In these instrumental learning tasks, animals initially lever press purposefully in order to obtain the outcome and will cease lever pressing once the food outcome is devalued. However, following extensive training animals will shift to habitual responding and will continue pressing the lever even after the food outcome has been devalued (40). As originally demonstrated in the plus-maze (36), the transition from cognition to habit in instrumental learning tasks might also be attributed to a neuroanatomical shift. The initial cognitive control of behavior in these instrumental learning tasks is mediated by the hippocampus and DMS (43, 44), whereas later habitual responding is mediated by the DLS (18, 45, 46).

Numerous investigators have suggested that the neuroanatomical shift to habit memory demonstrated in maze and instrumental learning tasks might also underlie the shift from recreational drug use to compulsive drug abuse (13, 47–50). Consistent with this hypothesis, investigators have demonstrated for a variety of abused substances that the DMS mediates goal-directed responding for drug reinforcement and the DLS mediates habitual responding for drug reinforcement (18, 31, 51–53).

Considering the high abuse potential of some drugs, investigators have suggested that addictive drugs might enhance DLS-dependent habit memory function and thereby accelerate the shift from cognitive to habitual control of behavior. Consistent with this hypothesis, repeated exposure to amphetamine or cocaine facilitates the shift from goal-directed to habitual responding for food reinforcement in instrumental lever pressing tasks (31, 54–59). In addition, lever pressing for addictive substances (e.g., alcohol or cocaine) versus food reward has been associated with greater habitual responding versus goal-directed responding (24, 60, 61). In humans, alcohol-dependent individuals show greater habitual responding in an instrumental learning task, relative to non-dependent control individuals (62). This enhancement of DLS-dependent habit memory by addictive drugs has also been observed in rodent maze learning tasks. Cocaine, amphetamine, and alcohol exposure have been associated with enhanced learning in DLS-dependent maze tasks or greater use of DLS-dependent response strategies in dual-solution versions of the maze (25, 63, 64). In humans, the use of abused substances, including alcohol and tobacco, has been correlated to the greater use of dorsal striatum-dependent navigational strategies in a virtual maze (65). Thus, some drugs of abuse might enhance DLS-dependent habit memory, and this heightened engagement of the DLS memory system might accelerate the transition from recreational drug use to habitual drug abuse. This proposed mechanism is consistent with White’s (1) original contention that drugs of abuse might sometimes facilitate their own self-administration by enhancing function of memory systems.

## Competition Between Memory Systems

Although it is possible that addictive drugs enhance habit memory directly by enhancing function of the DLS [e.g., Ref. (29)], another possibility is that drugs of abuse enhance habit memory indirectly via modulation of other memory systems. This alternative mechanism invokes the hypothesis that in some learning situations, memory systems compete for control of learning and that by impairing the function of one memory system, function of another intact system might be enhanced (11, 66). Notably, the hippocampus and DLS might sometimes compete for control of learning, whereby lesion of the hippocampus enhances DLS-dependent memory function (5, 6, 67, 68). Competitive interactions can also be demonstrated in dual-solution tasks, when impairing one memory system results in the use of a strategy mediated by another intact system. For instance, animals given DMS lesions display DLS-dependent habitual responding for food reward in instrumental learning tasks (44).

Considering the competitive interactions that sometimes arise between memory systems, one possibility is that some drugs of abuse might enhance DLS-dependent habit memory indirectly by impairing cognitive memory mechanisms mediated by the DMS and hippocampus. As noted previously, alcohol is associated with greater use of DLS-dependent habit memory in maze and operant lever pressing paradigms (24, 61, 62, 64, 65). Evidence also indicates that alcohol impairs learning in hippocampus-dependent spatial memory tasks [(64, 69–72); for review, see Ref. (73)], as well as in DMS-dependent reversal learning tasks (74–77). Consistent with a competitive interaction between memory systems, it has been hypothesized that alcohol may facilitate DLS-dependent habit memory indirectly via impairing cognitive memory mechanisms (78).

It should be noted that aside from alcohol, numerous drugs have been associated with cognitive memory deficits. Exposure to morphine, heroin, methamphetamine, MDMA (ecstasy), or chronic cocaine similarly produces hippocampus-dependent spatial memory impairments across a variety of tasks (79–89). It is tempting to speculate that, as suggested for alcohol, cognitive memory impairments produced by addictive drugs might indirectly enhance DLS-dependent habit memory, and that this might be one mechanism allowing drug self-administration to become habitual in human drug abusers. On the other hand, it is also possible that spatial learning deficits produced by addictive drugs might occur indirectly via enhancement of DLS-dependent memory processes. Consistent with this hypothesis, stimulating CREB activity in the DLS impairs hippocampus-dependent spatial memory (90), whereas inhibition of CREB activity in the DLS reverses the spatial memory impairments produced by morphine (91).

## Role of Stress and Anxiety

An additional consideration regarding the multiple memory systems approach to drug addiction is the role of stress. Converging evidence indicates that robust emotional arousal facilitates DLS-dependent habit memory in rodents and humans [for reviews, see Ref. (9–12)]. Administration of anxiogenic drugs enhances DLS-dependent response learning in the water plus-maze (92–97). This enhancement of DLS-dependent habit memory is also observed following exposure to unconditioned behavioral stressors [e.g., chronic restraint, tail shock, predator odor, etc. (98–101)] and exposure to fear-conditioned stimuli [tone previously paired with shock (102, 103)]. Although originally demonstrated in rodents (92), this enhancement of habit memory induced by robust emotional arousal has also been demonstrated extensively in humans (99, 104–110).

The mechanisms allowing stress/anxiety to facilitate habit memory remain largely unknown; however, evidence indicates a critical modulatory role of the BLA (93–95, 100). Consistent with a competitive interaction between memory systems, some evidence also suggests that stress/anxiety might enhance DLS-dependent habit memory indirectly by impairing hippocampal function (94, 95).

Enhancement of habit memory following stress or anxiety may be relevant to understanding some prominent factors leading to drug abuse. Namely, stressful life events or chronic prolonged periods of stress/anxiety are associated with increased vulnerability to drug addiction and relapse in humans (111–117), and similar observations have been made in animal models of drug self-administration [for review, see Ref. (118)]. Investigators have suggested that consistent with the influence of emotional arousal on multiple memory systems (10), acute or chronic stress may enhance drug addiction and relapse in humans by engaging DLS-dependent habit memory processes (9, 49, 119). Consistent with this suggestion, stress in cocaine-dependent individuals is associated with decreased blood-oxygen-level-dependent (BOLD) activity in the hippocampus and increased activity in the dorsal striatum, and these BOLD activity changes are associated with stress-induced cocaine cravings (120).

## Emerging Sources of Addiction

Aside from drugs of abuse, the multiple memory systems hypothesis has also been recently employed for understanding other emerging sources of addiction. For instance, the rise in obesity over the past few decades has led to a comparable surge in experimental interest, with many investigators drawing parallels between drug addiction and overeating [for review, see Ref. (121–123)]. Some recent evidence has suggested that like drug addiction, food addiction might be partially attributed to heightened engagement of DLS-dependent habit memory. In rats, binge-like food consumption facilitates the shift from cognitive to habitual control of behavior (124, 125). Moreover, habitual behavior in bingeing animals is associated with increased DLS activity and may be prevented by blocking AMPA or dopamine D1 receptors in the DLS (125). Diet-induced obesity has also been recently associated with the use of habit memory in a Y-maze task (126).

Another emerging behavioral disorder that parallels some features of drug addiction is pathological video game playing or video game addiction [for review, see Ref. (127)]. Like drug addiction, long-term excessive video game playing has been associated with reduced dopamine D2 receptor binding in the dorsal striatum (128). Videogame playing is also correlated to increased activation of the dorsal striatum (129, 130), and greater dorsal striatal volumes predict higher levels of video game skill (131). People who regularly play action video games are more likely to use dorsal striatum-dependent habit memory in a virtual maze (132), and pre-training video game playing leads to habitual responding over goal-directed responding in a two-stage decision-making task (133). Thus, as proposed for drugs of abuse, playing video games might enhance video game addiction via engaging the DLS-dependent habit memory system.

Finally, the multiple memory systems approach might also be useful for understanding marijuana addiction. Although marijuana may have lower abuse potential than other illicit substances classically considered within the context of drug addiction research (e.g., cocaine, morphine, heroin, etc.), heavy cannabis use can nevertheless promote drug dependence and withdrawal symptoms as observed with other drugs of abuse (134–137). It has recently been suggested that marijuana addiction might be partially attributed to increased engagement of DLS-dependent habit memory (138). Whereas acute cannabinoid exposure impairs DLS-dependent memory function (139, 140), repeated cannabinoid exposure leads to greater DLS-dependent habitual responding in an instrumental learning task (141). In addition, heavy cannabis users display greater activation of the dorsal striatum, relative to non-users, when performing a marijuana version of the implicit association task (142), and participants with a history of cannabis use are more likely to use dorsal striatum-dependent habit memory in the virtual maze (65).

Given the successful application of the memory systems approach to emerging sources of addiction, it is reasonable to hypothesize that multiple memory systems might also be implicated in other behavioral pathologies associated with addiction, such as compulsive shopping, Internet addiction, and sex addiction. Indeed, whether the memory systems approach might be useful for understanding pathological gambling has also received some attention (143, 144).

## Conclusion

Twenty years of experimental evidence has largely corroborated White’s (1) multiple memory systems approach to drug addiction. Evidence indicates that the hippocampus mediates contextual control of drug self-administration, the DLS mediates S–R habitual responding for drug reinforcement, and the amygdala mediates conditioned drug seeking. In addition, subsequent research has led to additional insights regarding the multiple memory systems view of drug addiction including the shift to habit memory, competition between memory systems, and the role of stress and anxiety.

Future research should attempt to integrate the memory systems approach with other theories of addiction, such as opponent motivational processes (145). It would also be useful to incorporate into the memory systems view additional features of addiction, such as drug dependence, tolerance, and withdrawal. Although the present review predominantly focused on the brain regions originally considered by White (i.e., the hippocampus, dorsal striatum, and amygdala), it should be noted that additional brain regions related to learning and memory have also been critically implicated in drug addiction and relapse, including the medial prefrontal cortex and nucleus accumbens [for review, see Ref. (13)]. Finally, although beyond the scope of the present review, it should be acknowledged that extensive evidence suggests that cellular and molecular changes in the midbrain dopaminergic system also contribute to addiction (146).

Although habit memories might be especially difficult to control, some evidence indicates that DLS-dependent memory, once acquired, can in some circumstances be suppressed (147) or even reversed (148, 149). Thus, it is possible that the pharmacological manipulations and behavioral procedures leading to the reversal or suppression of habit memory in animal models of learning might potentially be adapted to treat drug addiction and relapse in humans.

## Author Contributions

JG and MP both contributed ideas and writing of the present mini-review.

## Conflict of Interest Statement

The authors declare that the research was conducted in the absence of any commercial or financial relationships that could be construed as a potential conflict of interest.
